# Supplementary material for: Switchable Optical Properties of Dyes and Nanoparticles in Electrowetting Devices
Source: Nanomaterials (Basel). 2024 Jan 9;14(2):142. doi: 10.3390/nano14020142 (PMC10821281; doi:10.3390/nano14020142)
Supplement: Supplementary file 1 [file nanomaterials-14-00142-s001.zip › nanomaterials-2765160-supplementary.pdf]

Switchable Optical Properties of Dyes and Nanoparticles in Electrowetting Devices

Urice N. Tohgha [1,2], Jack T. Ly [3], Kyung Min Lee [1,2], Zachary M. Marsh [1], Alexander M. Watson [4], Tod A. Grusenmeyer [1], Nicholas P. Godman [1] and Michael E. McConney [1,*]

[1] Air Force Research Laboratory, Materials and Manufacturing Directorate, Wright-Patterson AFB, OH 45433, USA; urice.tohgha.ctr@us.af.mil (U.N.T.); kyungmin.lee.3.ctr@us.af.mil (K.M.L.); zachary.marsh.2.ctr@us.af.mil (Z.M.M.); tod.grusenmeyer.1@us.af.mil (T.A.G.); nicholas.godman.2@us.af.mil (N.P.G.)
[2] Azimuth Corporation, Fairborn, OH 45431, USA
[3] UES, Inc., Dayton, OH 45432, USA; jack.ly.ctr@us.af.mil
[4] Department of Engineering Management, School of Engineering, Systems, and Technology, University of Dayton, OH 45469, USA
* Correspondence: michael.mcconney.1@us.af.mil

Table of Contents

Fig S1. Transmittance spectrum of a blank control (300 x 900 µm pixel dimensions).
Fig S2. Transmittance spectra of device (300 x 900 µm pixel dimensions) dosed with dye **1** before actuation (0V) and after actuation (V OFF). Hysteresis observed.
Fig S3. Transmittance spectra of device (300 x 900 µm pixel dimensions) dosed with dye **2** before actuation (0V) and after actuation (V OFF). Hysteresis observed.
Fig S4. Transmittance spectra of device (300 x 900 µm pixel dimensions) dosed with dye **3** before actuation (0V) and after actuation (V OFF). Hysteresis observed.
Fig S5. Transmittance spectra of device (200 x 600 µm pixel dimensions) dosed with dye **3** showing no device response at 0-10 V.
Fig S6. Transmittance spectra of device (200 x 600 µm pixel dimensions) dosed with dye **3** showing three cycles of actuation and little to no hysteresis.
Fig S7. Transmittance spectra of device (150 x 450 µm pixel dimensions) dosed with dye **3** showing no actuation of device.
Fig S8. Absorption spectra of Ag nanoparticles (left), CdSe nanotetrapods (middle) and nanoplatelets (right) in solvent.
Fig S9. Transmittance spectra of device (300 x 900 µm pixel dimensions) dosed with **Ag** nanoparticles. Four actuation cycles show little to no hysteresis.
Fig S10. Transmittance spectra of device (300 x 900 µm pixel dimensions) dosed with **CdSe** nanoparticles. Different device used to further evaluate the optical properties of CdSe in devices.

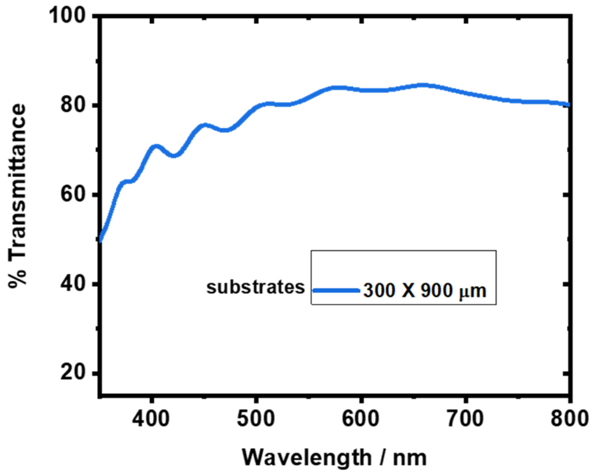

Figure S1. Transmittance spectrum of a blank control (300 x 900 µm pixel dimensions).

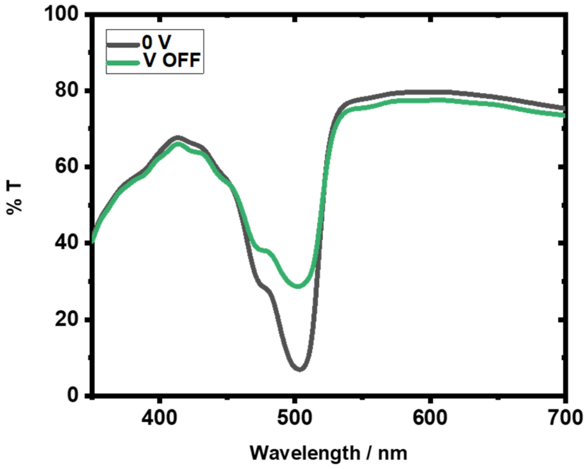

Fig S2. Transmittance spectra of device (300 x 900 μm pixel dimensions) dosed with dye **1** before actuation (0V) and after actuation (V OFF). Hysteresis observed.

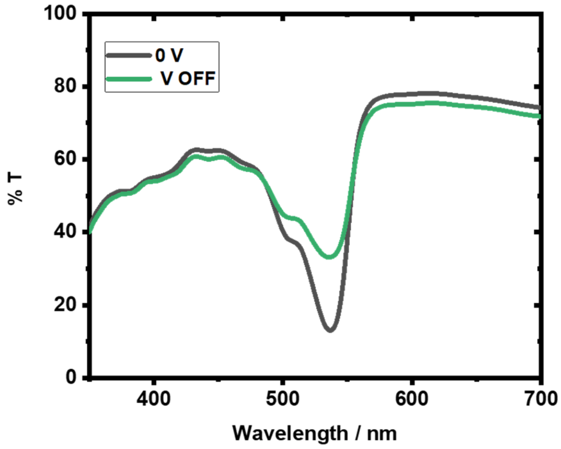

Figure S3. Transmittance spectra of device (300 x 900 μm pixel dimensions) dosed with dye **2** before actuation (0V) and after actuation (V OFF). Hysteresis observed.

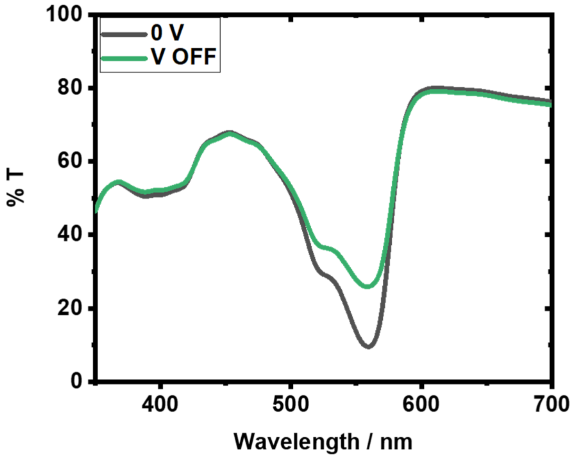

Figure S4. Transmittance spectra of device (300 x 900 μm pixel dimensions) dosed with dye **3** before actuation (0V) and after actuation (V OFF). Hysteresis observed.

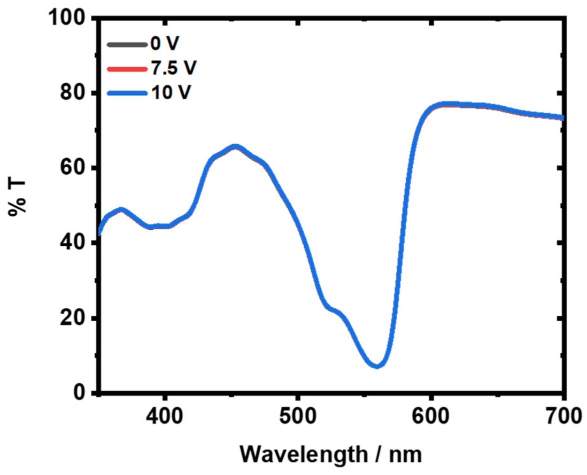

Figure S5. Transmittance spectra of device (200 x 600 μm pixel dimensions) dosed with dye **3** showing no device response at 0-10 V.

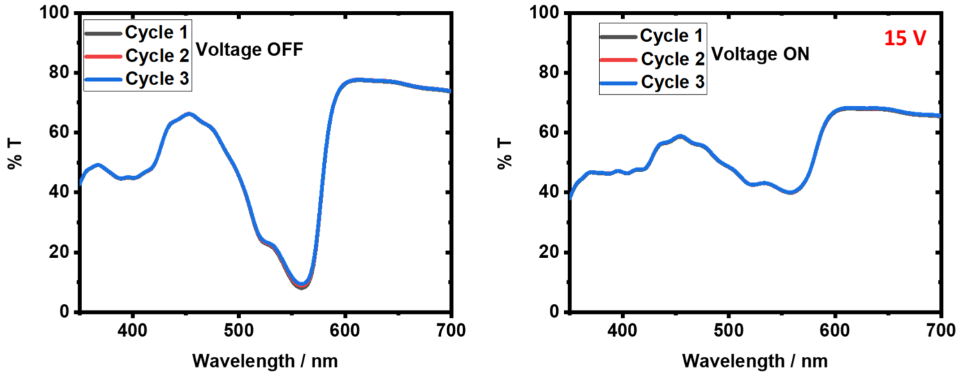

Figure S6. Transmittance spectra of device (200 x 600 μm pixel dimensions) dosed with dye **3** showing three cycles of actuation and little to no hysteresis.

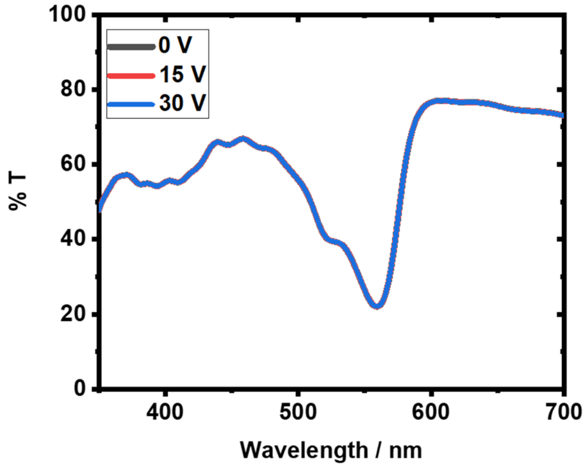

Figure S7. Transmittance spectra of device (150 x 450 μm pixel dimensions) dosed with dye **3** showing no actuation of device.

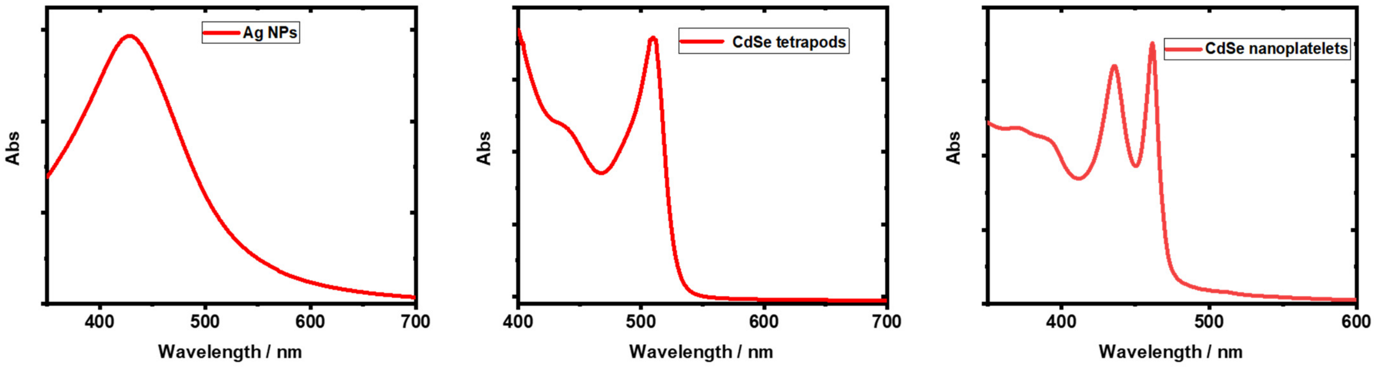

Figure S8. Absorption spectra of Ag nanoparticles (left), CdSe nanotetrapods (middle) and nanoplatelets (right) in solvent.

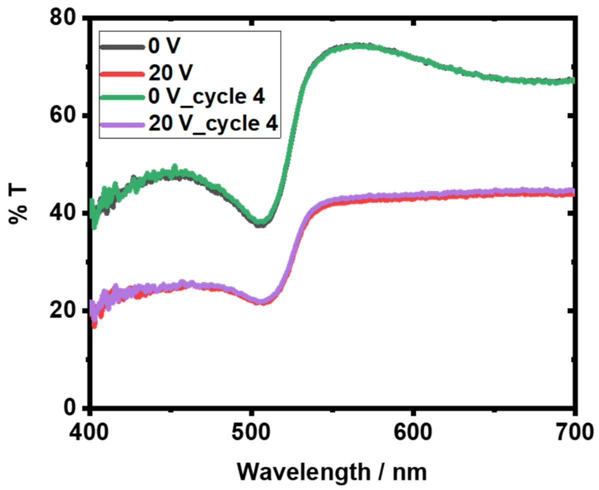

Figure S9. Transmittance spectra of device (300 x 900 μm pixel dimensions) dosed with **Ag** nanoparticles. Four actuation cycles show little to no hysteresis.

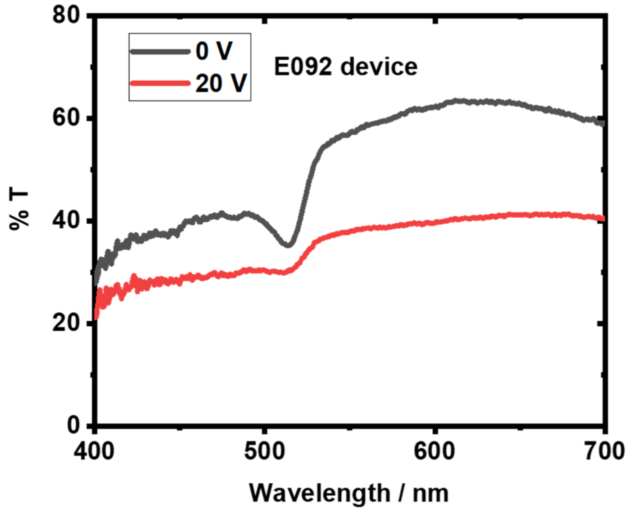

Figure S10. Transmittance spectra of device (300 x 900 μm pixel dimensions) dosed with **CdSe** nanoparticles. Different device used to further evaluate the optical properties of CdSe in devices.
